# Achalasia in the Elderly: Diagnostic Approach and a Proposed Treatment Algorithm Based on a Comprehensive Literature Review

**DOI:** 10.3390/jcm10235565

**Published:** 2021-11-26

**Authors:** Amir Mari, Wisam Sbeit, Wisam Abboud, Halim Awadie, Tawfik Khoury

**Affiliations:** 1 Gastroenterology and Endoscopy United, The Nazareth Hospital, EMMS, Nazareth 1613101, Israel; amir_mari@nazhosp.com; 2 Faculty of Medicine, Bar-Ilan University, Safed 5290002, Israel; wisams@gmc.gov.il (W.S.); wisam_abbud@nazhosp.com (W.A.); 3 Department of Gastroenterology, Galilee Medical Center, Nahariya 22100, Israel; 4 Department of Surgery, The Nazareth Hospital, EMMS, Nazareth 1613101, Israel; 5 Emek Medical Center, Institute of Gastroenterology and Hepatology, Afula 1855701, Israel; Halim_aw@clalit.org.il

**Keywords:** achalasia, elderly, approach, POEM, myotomy

## Abstract

Achalasia is not uncommonly diagnosed in elderly patients and its incidence and prevalence are growing in this population. However, a scarcity of studies has assessed the typical pathophysiological and clinical features of the disease as well as the effectiveness and safety of the various therapeutic options in elderly populations. Botulinum toxin injection has been used for achalasia treatment since 1994 and is traditionally considered the preferred treatment for fragile elder patients. However, recently more evidence has become available regarding the safety and effectiveness of pneumatic balloon dilation (BD), laparoscopic Heller myotomy (LHM) and per-oral endoscopic myotomy (POEM) in elderly patients with achalasia. In the current review we present the current literature on this topic with a focus on the clinical presentation of achalasia in the elderly and manometric features thereof, as well as summarize the effectiveness and safety of the various therapeutic options. Furthermore, we propose a practical management algorithm as a means to guide the treatment of future cases. We recommend that a conservative/BTI approach should be adopted in the fragile unfit patient. In the elderly fit patient, the treatment decision should be based on the achalasia type, patient preference and the available expertise, similar to the approach adopted for the non-elderly population.

## 1. Introduction

Achalasia originates from the Greek word *a-khalasis*, meaning lack of relaxation. The exact pathogenesis of achalasia is poorly known so far. Nevertheless, research findings propose a theory of an autoimmune reaction activated by a viral infection, leading to a cascade of a destructive inflammatory process resulting in destruction of the nitric oxide-releasing neurons within the myenteric plexus [1]. Achalasia is generally diagnosed between the third and sixth decades, and both genders appear to be similarly affected [2]. However, a study from in the Veneto Region of northeast Italy revealed that the incidence of achalasia among the elderly (>75 years old) was four times higher than in the younger population (<45 years old) [3]. Its presenting symptoms are classically dysphagia, chest pain, vomiting, and weight loss. The diagnosis of achalasia may be delayed for many years, thus a high level of clinical suspicion is needed. Achalasia is a chronic lifetime condition that profoundly disturbs patients’ quality of life and eventually may lead to severe nutritional difficulties, particularly among frail elderly patients. Despite the increasing prevalence of achalasia among elderly patients, only a few studies have assessed the clinical and the pathophysiological characteristics of the disease among elderly patients group. Some older studies have shown that both the sensory functions and visceral perception were significantly decreased with advanced age. The distinctive motility functions demonstrated in elder individuals include significant changes in esophageal peristalsis and delayed esophageal emptying when compared with younger age groups [4]. Other studies found a significant decrease in peristaltic wave amplitudes, as well as decreased pressures of esophageal contraction and reduction in the upper and lower sphincters’ pressures [5,6,7,8]. Autopsy studies have revealed a reduced number of ganglion cells in the myenteric plexus. Clouse et al. have studied the effect of aging on clinical presentation, manometric findings and outcomes among older patients (over 70) with achalasia compared with a younger patient’s group [9]. The main findings of the study that the disease presented similarly except that fewer older patients complained of chest pain as compared with the younger patients. Moreover, the only alteration observed was a lower residual pressure of the lower esophageal sphincter in elderly patients. A study by Schechter et al. that aimed to investigate clinical and manometric findings in older patients (over 60) compared with younger patients has revealed that elder achalasia patients are less symptomatic and have lower pressure of the lower esophageal sphincter (26.4 mm Hg vs. 31.9 mm Hg, *p* = 0.001) [10]. The target of achalasia treatment is to improve patients’ symptoms and their quality of life. The restoration of esophageal peristalsis is unrealistic; therefore, the eventual goal of therapy is to release the obstruction at the level of the EGJ. The decision regarding the optimal treatment choice essentially depends on the patient’s symptoms, comorbidities, age, achalasia subtype, preference, and the center expertise [11]. Botulinum toxin injection at the EGJ has been used for achalasia treatment since 1994 and is traditionally considered the preferred treatment for fragile elderly patients. However, recently more evidence has become available regarding the safety and effectiveness of pneumatic balloon dilation (BD), laparoscopic Heller myotomy (LHM) and per-oral endoscopic myotomy (POEM) in elderly patients with achalasia. Nonetheless, to this point, despite the high prevalence and disease burden in the elderly, the assessment, management and follow up of achalasia in this specific population is still a matter of debate. Remarkably, the Veneto study has shown that 60% of achalasia patients over 75 years of age were left untreated [3]. A possible reason for this surprising approach is the anticipated high risk of complications with endoscopic procedures or surgical interventions. In the current review we aim to provide an updated comprehensive review on the assessment and management of achalasia in elderly patients.

### Diagnostic Approach to Esophageal Dysphagia and Achalasia in the Elderly

The diagnostic approach to dysphagia and suspected achalasia in elderly patients does not differ from the younger patients’ groups. Dysphagia, per se, is considered an alarm symptom that mandates the performance of esophago-gastro-duodenoscopy (EGD) as an initial safe diagnostic modality to rule out any mechanical or anatomic obstruction in the esophagus or at EGJ such as infectious, tumors, inflammation, esophageal rings and strictures, and other pathologies [12]. Importantly, extra-esophageal compression and neurological and cerebrovascular etiologies should be sought. Classic endoscopic findings of achalasia present in about half of cases and include dilated esophagus, food and fluid contents, and obstructed EGJ. After the exclusion of anatomical, structural and inflammatory conditions, esophageal peristalsis and LES function should be assessed. HRM is the gold standard modality in investigating esophageal and LES functions and relies on the creation of a topographic spatiotemporal plot of esophageal peristalsis and LES function, where variations in pressure are represented as color changes over a time axis. The Chicago Classification is a practical scheme for analyzing and interpreting HRM studies as well as classifying esophageal motility into major and minor disorders [13]. The Chicago Classification subdivides achalasia into three subtypes. Achalasia type I has absent peristalsis, in type II the peristalsis is replaced by pan-esophageal pressurizations throughout the tubular esophagus, whilst type III is characterized by the presence of premature and/or spastic contractions. This subtyping has improved our understanding of achalasia and also impacts the therapeutic options therefor, permitting a tailored therapeutic strategy. Functional or idiopathic EGJ outflow obstruction (EGJOO)—Previously “variant achalasia”—Is characterized by the evidence of outflow obstruction at the level of the EGJ (characterized by defective LES relaxation), with intact esophageal peristalsis [13]. Recently, adjunctive tests are accompanied to HRM aiming to reproduce a more physiological drinking performance, using new tests, such as the rapid drinking challenge (RDC) and multiple rapid swallows (MRS) [14]. Barium esophagography has commonly been used to evaluate esophageal morphology prior to surgery. Recently, the timed barium swallow (TBS) has been used to assess treatment success by evaluating esophageal emptying. The barium column height and width at 1, 2 and 5 min have been used as an objective measure of the degree of LES obstruction, and post-treatment barium emptying has been shown to be a good objective predictor of treatment response [15]. TBS has several advantages; it is simple, practical, reproducible, economic, not invasive and well tolerated by patients. A recent study by Sanagapali et al. found that a change in barium surface area compared with that prior to therapy better correlates with treatment response when compared with the conventional post therapy barium column height at 5 min [16]. EndoFLIP is a novel diagnostic device permitting the quantification of EGJ distensibility and may be used to identify achalasia subtypes with a high level of sensitivity and specificity [17].

## 2. Treatment Options

### 2.1. Endoscopic Treatment

The endoscopic treatment of achalasia has reformed dramatically in recent years subsequent to the implementation of HRM for diagnosis and POEM for therapy. It is now well established that the preferred treatment of achalasia depends on the subtype determined by the HRM study. While types I and II may be treated by pneumatic dilatation, LHM or POEM, the preferred treatment for type III is POEM [18,19].

#### 2.1.1. Botulinum Toxin

Not far in the past, botulinum toxin injection was the most popular and commonly used due to its cost–efficacy profile and safety [20]. Most complications are mild, including chest pain, epigastric pain and heartburn [21], while serious complications are rare and include hepatic and sub-diaphragmatic abscesses [19]. During endoscopy, botulinum toxin A is injected into four to eight quadrants in the lower esophageal sphincter (LES). Despite an 80% clinical response, the major drawback is the short durability of response, with 60% recurrence within one year [22], with progressive weakening of treatment following repeated sessions. Botulinum toxin acts by preventing acetylcholine release from synapses, thus interrupting lower esophageal sphincter contractions; however, with repeated injections, normal acetylcholine release is restored, resulting in tachyphylaxis [23]. An early study showed the response rate to be greater in patients older than 50 years of age and in patients with vigorous achalasia [24]. Compared with other available treatments, botulinum toxin injection is regarded to be less effective; however, due to its safety and mini-invasiveness it should be reserved for the elderly, significantly comorbid patients unfit for other treatments or those unwilling to undergo other treatments. It also may be offered under primary failure or symptomatic recurrence after POEM or LHM [19]. Importantly, muscle-relaxant drugs such as nitric oxide, calcium channel blockers, or Sildenafil agents have generally limited efficacy, temporary effect, and are associated with adverse events such as headache, edema and dizziness [25].

#### 2.1.2. Pneumatic Dilation

Pneumatic dilation (PD) is more effective with a more durable response compared to botulinum toxin injection. Despite the need for repeated dilations, it is considered the most cost effective [26], and the most frequently adopted therapy for achalasia worldwide [3]. PD is contraindicated in patients with poor cardiopulmonary status or other comorbidities preventing surgery, should an esophageal perforation occur [26]. PD achieves its effect by tearing the LES, dilating its muscle fibers forcefully with an air-filled balloon [26]. A 2–4-weeks interval of successive dilations using increasing balloon sizes is usually needed, based on symptoms relief coupled with repeated LES pressure measurements or assessment of esophageal emptying improvement to objectively assess treatment response [3]. Minor complications occur in up to 33% of patients including chest pain, bleeding, mucosal tear without perforation, esophageal hematoma, transient fever, and aspiration pneumonia, while about 2% of patients may suffer from a potentially life-threatening esophageal perforation [27]. The success rate of PD may reach 70 per cent with a single dilatation, increasing to more than 90 per cent with multiple dilations [28]. However, about one-third of patients have recurrence of their symptoms within 4 to 6 years [18]. A retrospective study from England, including 6938 achalasia patients treated by PD or surgical Heller’s myotomy showed that mortality after PD was associated with increasing age, comorbidities, previous Heller’s myotomy and repeated PD [29]. On their multicenter survey of patients over 80 years of age, Zotti et al. showed that most specialized centers do not tailor treatment based on age, but on their physiological and mental health, and that endoscopic treatment has a high recurrence rate [30].

#### 2.1.3. Per Oral Endoscopic Myotomy (POEM)

POEM is the endoscopic equivalent of surgical myotomy. It was first introduced by the Japanese achalasia group and presented by Inoue et al. in 2009 [31]. Since then, POEM has been widely adopted internationally by interventional endoscopists as an alternative therapy to surgical myotomy. In addition, POEM was performed for other foregut disorders, such as diffuse esophageal spasm (DES) and gastroparesis. POEM is undertaken while then patient is supine and under general anesthesia. A mucosal incision is performed at 10–12 cm proximal to gastro-esophageal junction (GEJ) to facilitate submucosal tunneling followed by circular muscle bundle (or full thickness) myotomy of a total length of 8 cm (6 cm in the lower esophagus and 2 cm in the cardia) [31]. POEM is considered a primary treatment for type I and type II achalasia and it is the ideal treatment option for longer segment disorders of type III achalasia. Although POEM is a relatively a safe procedure, the contraindications to POEM are usually the same as surgical contraindications. Prior interventional therapies (Botox injection, pneumatic dilation, Heller’s myotomy) are not considered contraindications to POEM; however, a selective approach is needed in such cases. Age is not a limitation in patient selection for POEM procedure. POEM has been successfully performed in both children and the elderly, as previously shown [32,33,34,35]. Inoue et al. reported the first case series of 17 patients with achalasia treated with POEM [31]. This first report showed significant reduction in dysphagia symptom score (10 to 1.3; *p* = 0.0003) and marked improvement in lower esophageal sphincter (LES) pressure (52.4 mmHg to 19.9 mmHg, *p* = 0.0001). The short-term outcome (5 months) showed excellent results, and this was followed and confirmed by other international studies. A multicenter prospective single-arm Japanese study was published by Shiwaku et al. [36], reporting on 233 patients with achalasia who underwent POEM with 97.4% clinical success as defined by an Eckardt score (ES) <3 in 1 year. Post-POEM reflux esophagitis, severe reflux esophagitis and symptomatic GERD were 54.2%, 5.6% and 14.7%, respectively. PPI was administered in 21.1% of patients [34]. A systematic review by Evensen et al. [37] reported on 355 treatment-naive patients who had POEM from different studies published during the last ten years showing >90% clinical success [37]. Endoscopic myotomy can be performed on the anterior or posterior wall of the esophagus. Anterior myotomy was the main approach; later, a posterior myotomy was reported. Khashab et al. reported an RCT of 150 achalasia patients who received either anterior (73) or posterior (77) myotomy [38]. Of them, 138 completed a 1-year follow up, and technical success was achieved in 97.3% and 100% of the anterior and posterior groups, respectively. Clinical success was 90% and 89% in the anterior and posterior groups, respectively. Adverse events were comparable between the two groups [38]. A recent report on two-years follow ups showed that both techniques remained equally effective and GERD outcomes were also similar [39]. An RCT reported on 63 patients who were randomized to either anterior (31 patients) or posterior (33 patients) groups. The short-term treatment efficacy, manometry outcomes and adverse events were comparable between both groups [40]. When POEM is performed following previous LHM, posterior myotomy is suggested and preferred due to previous myotomy on the anterior wall performed earlier, during LHM. Unless a selective approach indicated, it is mainly an endoscopist preference. Moreover, one hundred thirty patients were randomized to receive either POEM or pneumatic dilatation (PD) (64 vs. 66 patients, respectively) [41]. Clinical success (ES < 3 at 2 years) was 92% and 54% in the POEM and PD groups, respectively (*p* < 0.01). Reflux esophagitis was reported in 41% of the POEM group and 7% of the PD group (*p* = 0.002) [41]. Werner et al. have reported an international, multicenter, randomized controlled study comparing POEM vs. laparoscopic Heller’s myotomy (LHM) with Dor’s fundoplication for symptomatic achalasia [42]. They showed a long term (two years follow up) clinical success in both POEM and LHM (83% vs. 81.7%, *p* = 0.0001 for noninferiority). Serious adverse events occurred in 2.7% of patients in the POEM group and 7.3% of patients in the LHM group. At 3 months and 24 months following intervention, reflux esophagitis was more common in the POEM group vs. LHM group (57% vs. 20% and 44% vs. 29%, respectively) [42]. Notably, both surgical and endoscopic myotomy are associated with post-myotomy reflux characterized by gastroesophageal reflux disease and reflux esophagitis (RE), which are the main and most common side effects of post-myotomy treatment. Both GERD and RE are more frequent after POEM than after LHM with fundoplication. Post POEM GERD is usually asymptomatic and those who are symptomatic have responded to PPI [43]. A systematic review by Repici et al. has reported a pooled rate of 29% of RE after POEM and 7.6% after LHM [44]. This difference was also reported by Werner et al., as shown above. Hence, RE is still the main post-POEM side effect but it is less reported following LHM due to the fundoplication effect. Lately, POEM + fundoplication (POEM+F) was reported as an adjunctive endoscopic approach to prevent post-POEM reflux [45]. This was reported by Inoue et al. who reported on 21 patients with successful endoscopic procedures. An endoscopic wrap mimicking laparoscopic fundoplication was seen in all patients following the procedure at up to two months follow up. The clinical course post-POEM+F was uneventful; however, the clinical success preventing GERD was not reported. To date, PPI is the treatment of choice in post POEM GERD/RE. Minimal invasive myotomy (MIM) was reported in 57 elderly (age 78, mean) with achalasia, showing the surgery was feasible, but not without complication (19.3%) [46]. Clinical success was reported in 96.5% of cases and the improvement of dysphagia score ranged between 3.38 and 1.36 in all patients. Observational studies have reported on POEM in the elderly. A Chinese group led by Li et al. reported on 15 patients with achalasia older than 65 years (65–84) [33]. Clinical success, defined as an Eckardt score of ≤3, was 100% and no major complication was related to the procedure. Another study by Tang et al. retrospectively reported the efficacy of POEM in 18 patients older than 60 (60–74) years and in 95 patients younger than 60 years (18–59) [34]. They found that the efficacy was 92.9% and 89.9% respectively, (*p* = NS), and no difference in the rate of adverse events in both groups (*p* = NS) was observed. POEM and pneumatic balloon dilatation in patients older than 65 years (67.9 +/− 4.3) was reported [35]. The efficacy of these two procedures was 92.2% and 80.0%, respectively, without any major complications. AEs, mainly GERD/RE are treatable. During the last 12 years, POEM was performed in all age groups, including the elderly; however, these were observational studies. Given the minimal invasive approach in POEM compared with surgical intervention, it is considered safe and feasible. Finally, as stated in the clinical practice guidelines for POEM by the Japanese gastroenterological endoscopy society [47], POEM is an effective and safe procedure for elderly patients. Future studies are needed to assess the feasibility and safety of POEM in elderly.

## 3. Surgical Treatment

### Laparoscopic Heller Myotomy in Elderly Patients

Laparoscopic Heller myotomy (LHM) was introduced by Ernst Heller in 1913. Modified laparoscopic Heller myotomy was introduced in 1923 and includes anterior cardiomyotomy, in which the esophageal myotomy should be at least 5 cm long. Myotomy is related to the mechanical dissection of the anterior muscle fibers. It is acceptable, for the purpose of preventing post treatment reflux, to perform a partial fundoplication wrap, either posteriorly (toupet) or anteriorly (dor). The success rate, measured by symptom improvement scores, following LHM is estimated to be 85% after 5 years [48]. Until recently, laparoscopic Heller myotomy (LHM) was considered to be the gold standard and proved to ensure satisfactory, long-term symptomatic relief along with low complications rate. Moreover, LHM also permits the surgeon to fashion an anti-reflux barrier in order to minimize subsequent reflux [49,50]. The place of LHM in terms of efficacy and safety among elderly achalasia patients has not been studied in high-quality, prospective clinical trials. Nonetheless, few comparative studies have assessed this important question. Randal et al. studied, in a retrospective study, the effectiveness (symptoms-based) and the safety of LHM in 51 elderly patients (mean age 73.14, range 65–89 years). The intra- and perioperative complications were very low; one patient had gastric complication and one patient had lung atelectasis. No mortality cases were reported. The average length of hospital stay was 3 days. All 42 patients who completed a follow-up (mean time 42 months, range 24 to 53), all reported overall dysphagia symptoms relief. The authors concluded that being of elderly age does not appear to negatively impact LHM outcomes [51]. An interesting Italian study by Renato et al. evaluated the effect of age on LHM in over 571 achalasia patients divided into three age groups; A (≤45 years), B (45–70), and C (≥70) [52]. Notably, the older patients (group C) had more symptoms at preoperative assessment. The operation-related mortality was nil in all groups. Post-operative complications occurred only in one patient from group C (bleeding from the myotomy edge, which was managed conservatively). Hospital stay was relatively longer for group C compared with the other groups (*p* = 0.06). At a median follow-up of 38 months symptom scores were significantly lower after surgery, unrelatedly with patient’s age. Likewise, Arman et al. have published their experience with minimally invasive Heller myotomy in elderly patients [46]. A total of 57 patients with a mean age of 78 years (range 70 to 96 years) were included. There was no perioperative mortality and the median hospital stay was 3 days. The complications rate was low and included three intraoperative esophageal perforations, three pleural effusions, two cases of hospital-acquired infections, one case of gastric perforation and two cases of post-operative ileus. All patients with complications were managed conservatively and no mortality cases were reported. The mean follow-up period was 23.5 months. All patients had improved dysphagia scores. The authors concluded that the minimally invasive approach is effective and safe in old patients and should be considered a first-line therapy in surgically fit patients. A multicenter international survey that aimed to assess decision making on achalasia treatment and patients’ clinical outcomes in patients older than 80 years included seven centers (three from Brazil, three from the USA and one from Italy). Overall, 85 patients (mean age 84 ± 4) were included. LHM was performed in 20 patients with good clinical response and a very low complications rate. One major highlight from this survey is the fact that physicians from experienced high-volume centers did not mould treatment solely based on advanced age and proposed that LHM appears to be a reasonable therapeutic option, with good clinical outcomes and safety profiles, in this population [30]. Put all together, based on the data presented here and counting the enormous worldwide experience with LHM in elderly patients, this option seems to be practical, effective and safe in elderly fit patients. Nonetheless, published data in this population were mainly retrospective comparative studies and no randomized controlled trails could be found in the published literature, yet. Furthermore, more aspects should be studied while managing elderly achalasia patients, including the impact of achalasia subtype on the various therapeutic options outcomes, along with long term clinical outcomes measured with objective tools like fluoroscopy, manometry and EndoFlip.

## 4. Conclusions and a Proposed Approach

**Figure 1:**
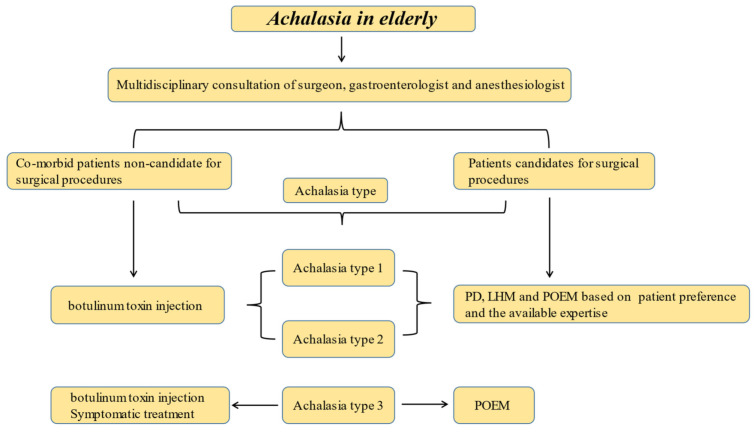
A proposed algorithm for the management of achalasia in the elderly.

To summarize, achalasia’s incidence and prevalence are growing in the elderly population, probably due to a noticeable delay in diagnosis. Based on the current knowledge, no hallmark clinical or manometric features are specific to this population. Furthermore, based on the current knowledge, pneumatic dilation, LHM and POEM seems to have satisfactory effectiveness and safety profiles. We suggest (Figure 1) that botulinum toxin is a reasonable approach in the fragile unfit patient. In fit, elderly patients, the treatment decision should be based on the achalasia type, patient preference and the available expertise, similar to the approach adopted for the non-elderly population. A randomized prospective study is needed to further characterize the best treatment modality in elderly patients.

## Abbreviations

LES	Lower esophageal sphincter
EGJ	Esophago-gastric junction
EGJOO	Esophago-gastric outflow obstruction
HRM	High-resolution manometry
POEM	Per oral endoscopic myotomy
EoE	Eosinophilic esophagitis
EGD	Esophago-gastro-duodenoscopy
LHM	Laparoscopic Heller myotomy
RDC	Rapid drinking challenge
MRS	Multiple rapid swallows
TBS	Timed barium swallow
